# A real-world data analysis of Clinical Global Impression-Severity (CGI-S) as a transdiagnostic predictor of psychiatric hospitalisation

**DOI:** 10.1192/j.eurpsy.2023.1290

**Published:** 2023-07-19

**Authors:** E. Palmer, M. Taquet, K. Griffiths, S. Ker, C. Liman, S. N. Wee, S. Kollins, R. Patel

**Affiliations:** 1 Holmusk, New York, United States; 2 University of Oxford, Oxford; 3 Institute of Psychiatry, Psychology & Neuroscience, King’s College London, London, United Kingdom

## Abstract

**Introduction:**

Preventing psychiatric admissions holds benefits for patients as well as healthcare systems. The Clinical Global Impression-Severity (CGI-S) scale is a 7-point measurement of symptom severity, independent of diagnosis, which has shown capability of predicting risk of hospitalisation in schizophrenia. Due to its routine use in clinical practice and ease of administration, it may have potential as a transdiagnostic predictor of hospitalisation.

**Objectives:**

To investigate whether early trajectories of CGI-S scores predict risk of hospitalisation over a 6 month-follow-up period.

**Methods:**

A retrospective cohort study was conducted, analysing Electronic Health Record (EHR) data from the NeuroBlu Database (Patel et al. BMJ Open 2022;12:e057227). Patients were included if they had a psychiatric diagnosis and at least 5 recorded CGI-S scores within a 2-month period, defined as the ‘index’ period. The relationship between early CGI-S trajectories and risk of hospitalisation was investigated using Cox regression. The analysis was adjusted for age, gender, race, number of years in education, and psychiatric diagnosis. Early CGI-S trajectories were estimated as clinical severity (defined as the mean CGI-S score during the index period) and clinical instability (defined as a generalised Root Mean Squared Subsequent Differences of all CGI-S scores recorded during the index period). The primary outcome was time to psychiatric hospitalisation up to 6 months following the index period. Patients who had been hospitalised before or within the index period were excluded.

**Results:**

A total of 36,914 patients were included (mean [SD] age: 29.7 [17.5] years; 57.3% female). Clinical instability (hazard ratio: 1.09, 95% CI 1.07-1.10, p<0.001) and severity (hazard ratio: 1.11, 95% CI 1.09-1.12, p<0.001) independently predicted risk of hospitalisation. These associations were consistent across all psychiatric diagnoses. Patients in the top 50% of severity and/or instability were at a 45% increased risk of hospitalisation compared to those in the bottom 50% (Figure 1).

**Image:**

**Figure:**
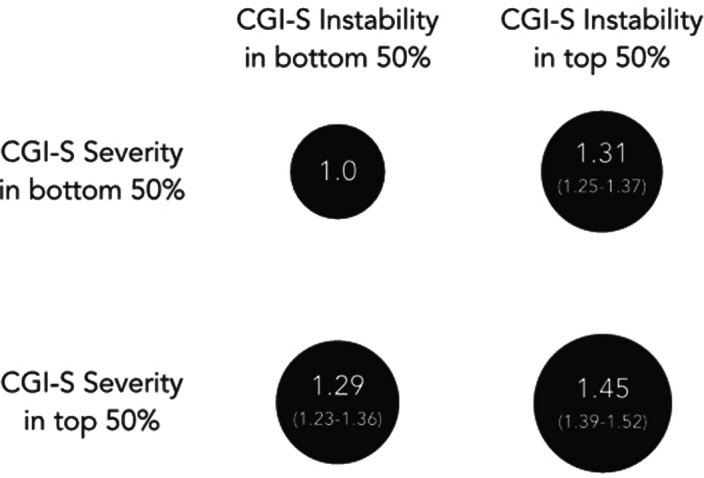

**Conclusions:**

Early CGI-S trajectories reflecting clinical severity and instability independently predict risk of hospitalisation across diagnoses. This risk was compounded when instability and severity were present together. These results have translation potential in predicting individuals who are at high risk of hospitalisation and could benefit from preventative strategies to mitigate this risk.

**Disclosure of Interest:**

E. Palmer Employee of: Holmusk, M. Taquet Consultant of: Holmusk, K. Griffiths Employee of: Holmusk, S. Ker Employee of: Holmusk, C. Liman Employee of: Holmusk, S. N. Wee Employee of: Holmusk, S. Kollins Employee of: Holmusk, R. Patel Grant / Research support from: National Institute of Health Research (NIHR301690); Medical Research Council (MR/S003118/1); Academy of Medical Sciences (SGL015/1020); Janssen, Employee of: Holmusk

